# Assessing the validity of the Self versus other interest implicit association test

**DOI:** 10.1371/journal.pone.0234032

**Published:** 2020-06-01

**Authors:** Emily M. Thornton, Lara B. Aknin

**Affiliations:** Department of Psychology, Simon Fraser University, Burnaby, British Columbia, Canada; Middlesex University, UNITED KINGDOM

## Abstract

There is great variability in the ways that humans treat one another, ranging from extreme compassion (e.g., philanthropy, organ donation) to self-interested cruelty (e.g., theft, murder). What underlies and explains this variability? Past research has primarily examined human prosociality using explicit self-report scales, which are susceptible to self-presentation biases. However, these concerns can be alleviated with the use of implicit attitude tests that assess automatic associations. Here, we introduce and assess the validity of a new test of implicit prosociality–the Self versus Other Interest Implicit Association Test (SOI-IAT)–administered to two samples in pre-registered studies: regular blood donors (Study 1; *N* = 153) and a nationally representative sample of Americans (Study 2; *N* = 467). To assess validity, we investigated whether SOI-IAT scores were correlated with explicit measures of prosociality within each sample and compared SOI-IAT scores of the control sample (representative sample of Americans) with the prosocial sample (blood donors). While SOI-IAT scores were higher in the prosocial blood donor sample, SOI-IAT scores were generally uncorrelated with explicit measures and actual prosocial behaviour. Thus, the SOI-IAT may be able to detect group differences in everyday prosociality, but future testing is needed for a more robust validation of the SOI-IAT. These unexpected findings underscore the importance of sharing null and mixed results to fill gaps in the scientific record and highlight the challenges of conducting research on implicit processes.

## Introduction

According to Giving USA Today, 2017 saw more money donated to charity than ever before, at $410 billion dollars [1]. That same year, there were nearly as many mass shootings as there were days in a calendar year, at 346 incidents [2]. While these statistics are limited to the United States, the contradictory picture of humanity they paint illustrates a broader paradox of human behaviour: on one hand, people donated more money than ever, and yet, at the same time, were extremely antisocial. This exemplifies a fundamental issue in social psychology and beyond: There is great variability in how humans treat each other, but how can we quantify and predict it? In two studies, we evaluate the construct validity of a novel method of prosocial attitude assessment that circumvents many of the pitfalls of traditional measures of prosociality–the Self versus Other Implicit Association Test (hereafter, SOI-IAT).

### Prosociality and measurement

Prosociality is commonly defined as any behaviour that is beneficial to other people [3]. While prosociality may appear maladaptive because it focuses attention and aid to others as opposed to oneself, evolutionary theorists have posited that altruism (and thus, prosociality) was essential to the evolution of group cooperation and by extension, to human survival (e.g., [4]). In this way, prosociality was of central importance to humanity’s cultural development and may underlie much of our social behaviour.

Given its importance, researchers from many disciplines have sought to measure and predict human prosociality. The most common method for doing so is with the use of explicit self-report scales, which are fast, easy, and convenient. Despite these valuable qualities, self-report scales are also vulnerable to *self-presentation*, which is when a respondent intentionally or unintentionally alters their response to present themselves in a favorable light [5]. Self-presentations concerns are especially relevant in the realm of morally laden topics, such as prosociality, because people may feel pressure to present themselves in a highly favourable manner [6,7]. As a result, the goal of the present research is to validate a new measure of implicit prosociality that can help to circumvent this limitation.

### Implicit attitudes and their assessment

A large body of research on social attitudes indicates that insight into an individual’s beliefs can be obtained by investigating a person’s implicit and/or explicit attitudes [5]. While both reflect an individual’s beliefs, explicit and implicit attitudes may differ. In line with recent recommendations urging researchers to indicate and define their use of implicit attitudes [8], we refer to implicit attitudes as automatically activated, quick, gut-level type evaluations [9]. This is in contrast to explicit attitudes, which are conscious and easily accessible [9]. To illustrate this difference, an individual may say they like math (an explicit positive attitude) but when faced with a math problem, they feel an instinctive sense of aversion (an implicit negative attitude). In this way, our conceptualization of implicit and explicit attitudes is most aligned with dual-process theory, which distinguishes between fast, automatic thinking (System 1) and slow, controlled thinking (System 2; [10]). Importantly, explicit attitudes are more easily captured using self-report measures, while implicit attitudes have historically been more difficult to measure because individuals may be unaware of their content [9].

One popular way to assess implicit attitudes is using the Implicit Association Test (IAT), originally introduced in 1998 by Greenwald, McGhee, and Schwartz. With the IAT, implicit attitudes are accessed by comparing the time respondents take to classify relevant words into dichotomous categories. According to the IAT framework, respondent’s word categorizations will be faster when the categorization is compatible with the respondent’s implicit attitude, and slower when the categorization is incompatible with their implicit attitude [11]. Compared to other measures of implicit attitudes, the IAT framework offers relatively efficient access into participant’s unconscious prosocial attitudes, with an attenuated risk of self-presentation effects [11].

Since its conception, the IAT has offered insight into implicit attitudes. For instance, using the IAT, researchers have discovered that large differences can exist between explicit and implicit attitudes in some domains [11]. As an example, one study found that participants who explicitly rejected prejudiced attitudes toward African Americans showed implicit prejudice levels similar to individuals who admitted to having prejudiced attitudes [11]. In other words, participants who explicitly reported holding non-prejudiced beliefs showed patterns of responding that aligned with prejudiced implicit attitudes. This demonstrates the potential for explicit-implicit attitude divergence and shows how certain testing methods, such as the IAT, can provide insight into previously inaccessible implicit attitudes.

Assessing implicit attitudes may also be an elegant solution to the challenges posed by explicit measures of prosociality in at least two ways. First, implicit attitudes are unlikely to be altered by self-presentation concerns because implicit attitudes are automatic and driven by gut reactions [12]. Second, most explicit measures of prosocial attitudes and behavior capture the degree to which an actor engages in other-oriented behavior or holds a positive attitude towards helping others on a spectrum ranging from low (absent or rare action/attitude) to high (common action/attitude). To illustrate, the Altruistic Personality Scale quantifies trait altruism as the frequency with an individual has engaged in altruistic acts on a scale from 1–7 [13], and the Prosocial Personality Battery assesses helpfulness and other-oriented empathy on a scale from 1–5 [14]. While valuable, this range does not include the opposite side of the “continuum of care”–self-interest [15]. The design of the IAT (described in more detail below) may be capable of accessing both other-interested and self-interested dimensions of social attitudes along a continuum, allowing researchers valuable insight into the full range of other-oriented attitudes.

### The Self versus other interest implicit association test

Given the limits of explicit measures of prosociality and the potential strengths of implicit attitude measures, such as the IAT, the present research offers a first look at the construct validity of a new measure of implicit prosocial attitudes, the Self versus Other Interest Implicit Association Test (SOI-IAT). The SOI-IAT is a new application of the IAT that assesses participant’s implicit attitudes toward two dichotomous categories: (1) *“Other Interest”* an implicit preference for benefitting others over oneself, and (2) *“Self Interest”* an implicit preference for benefitting oneself over others. In the SOI-IAT, individuals who are higher in “Other-Interest” should classify prosocial concepts (e.g., give) with positive information (e.g., wonderful) more quickly than they classify concepts of self-benefit (e.g., profit) with positive information. Meanwhile, people higher in “Self-Interest” should show the reverse, classifying self-benefit concepts with positive information faster than prosocial concepts with positive information. To assess these attitudes, participants’ self or other leaning was quantified as a *d-*score, a single output measure from the SOI-IAT that quantifies the direction and strength of one’s attitudes as measured by their categorization performance [16]. In the present research, positive *d-*scores indicate other-interest or prosociality, and negative *d-*scores indicate self-interest. Further, the more extreme (positive or negative) the *d-*score, the stronger the implicit attitude.

### The SOI-IAT versus the Pro-IAT

To our knowledge, the only other measure that has attempted to assess implicit prosocial attitudes is Marvel and Resh’s Prosocial-IAT (Pro-IAT; [17]). The Pro-IAT was designed to investigate a respondent’s implicit prosocial or self-serving attitudes by assessing implicit self-concepts. Specifically, Marvel and Resh (2018) investigated whether participants were faster to associate the target categories “Service” and “Profit” with the concepts “Me” and “They”. The authors of the Pro-IAT reasoned that individuals who were faster when “Service” and “Me” were paired likely include prosociality in their self-concepts, while speed when “Profit” and “Me” were paired was reflective of a “self-regarding, profit-driven orientation” ([17], p.10).

While at first glance, the SOI-IAT may resemble the Pro-IAT, further investigation reveals several critical differences. Firstly, Marvel and Resh (2018) developed the Pro-IAT for use in business and workplace psychology. As a result, the Pro-IAT’s conception of prosociality is workplace oriented, using target concepts “Service” and “Profit”. This focus is also illustrated by the fact that Marvel & Resh’s (2018) explicit measure of prosociality is comprised of items like “I want to help others through my work” and “It is important to me to do good for others through my work.” By contrast, in the present research we view prosociality as a broader construct that occurs beyond the workplace. For example, the explicit measures used in the present research include items such as “How often have you helped an acquaintance move households?” (Altruistic Personality Scale; [13]). Second, while the Pro-IAT assesses the content of an individual’s self-concept, or one’s perception of the self [17], the SOI-IAT assesses an individual’s implicit preferences. For example, while the Pro-IAT provides insight into how self-interested an individual implicitly considers themselves to be, the SOI-IAT reveals one’s implicit preference for helping others or helping themselves. As such, the SOI-IAT and Pro-IAT measure different facets of prosociality.

### The present research

In the present research, we assessed the construct validity of a new implicit measure of prosociality, the SOI-IAT, by investigating how SOI-IAT scores relate to explicit self-report measures of prosociality (convergent validity) and actual rates of helping behaviour (predictive validity) in two samples. In Study 1, we assessed the SOI-IAT’s validity using a sample of Canadian blood donors from Canadian Blood Services, and in Study 2, we assessed the SOI-IAT’s validity amongst a nationally representative sample of United States citizens. In a final analysis, we compared the mean SOI-IAT scores from the Canadian blood donor and American national panel samples to investigate whether the SOI-IAT could distinguish between the two groups. Data was collected cross-nationally for both practical and logistical reasons.

## Study 1: Blood donors study

### Methods

#### Participants

154 blood donors were recruited from a Canadian Blood Services location in Vancouver, Canada between February and May 2018. Consistent with our pre-registered data analysis plan, one participant was excluded from analyses because they were detected to be button mashing, (i.e., responded so quickly that an algorithm determined that they were likely answering randomly). This resulted in a total of 153 participants (*M*_age_ = 41.7, *SD* = 15.5, range = 19–77, 58 female, 84 male, 2 preferred not to say). In exchange for their time, participants could select a $10 Starbucks gift card or make a $10 donation to Canadian Blood Services. The full pre-registration, listing hypotheses, the data analysis plan, and exclusion criteria, can be found here.

#### Procedure

Participants were invited to complete a computer-based survey before or after their blood donation appointment. If interested, participants were instructed to find a research assistant at a quiet but public table near the post-donation resting area. There, the research assistant provided participants with a laptop computer showing an online survey and asked participants to follow the on-screen instructions.

After providing consent, participants completed a one-item measure of baseline happiness by reporting the extent to which they agreed with “I feel happy right now” on a 5-point Likert scale ranging from “1-Not at all” to “5-Extremely”. Next, participants completed a one-item baseline alertness measure by reporting the extent to which they agreed with “I feel alert right now”, on a 5-point Likert scale ranging from “1-Not at all” to “5-Extremely”. Afterward, participants completed the SOI-IAT (described below).

### SOI-IAT

#### Practice blocks

To assess their implicit prosociality, participants then completed the SOI-IAT. Like most Implicit Attitude Tests, the SOI-IAT has four counterbalanced sections and begins with two practice blocks (i.e., [11]). Because these blocks are designed to allow participants to become familiar with the SOI-IAT (i.e. present a “warm-up”), scores from the practice block are not used in analyses. In these blocks, participants hit specific keys to classify attribute words from the middle of the screen into one of two target categories in the upper corners of the screen. Importantly, there was only one correct classification for each word–if participants misclassified a word, they were shown a red ‘X’ and had to reclassify the word before continuing. In the SOI-IAT, the first practice block involved classifying the five concept-related words “Share”, “Give”, “Aid”, “Favor”, and “Assistance” into the “Other-Interest” category, and the five concept-related words “Keep”, “Gain”, “Earn", “Profit”, and “Obtain” into the "Self Interest” category. In the second practice block, participants classified pleasant and unpleasant words into corresponding categories, such as classifying “Beautiful” into the category “Good”, and “Terrible” into the category “Bad”. Participants were instructed to make these categorizations as fast as possible.

#### Critical blocks

**Fig 1:**
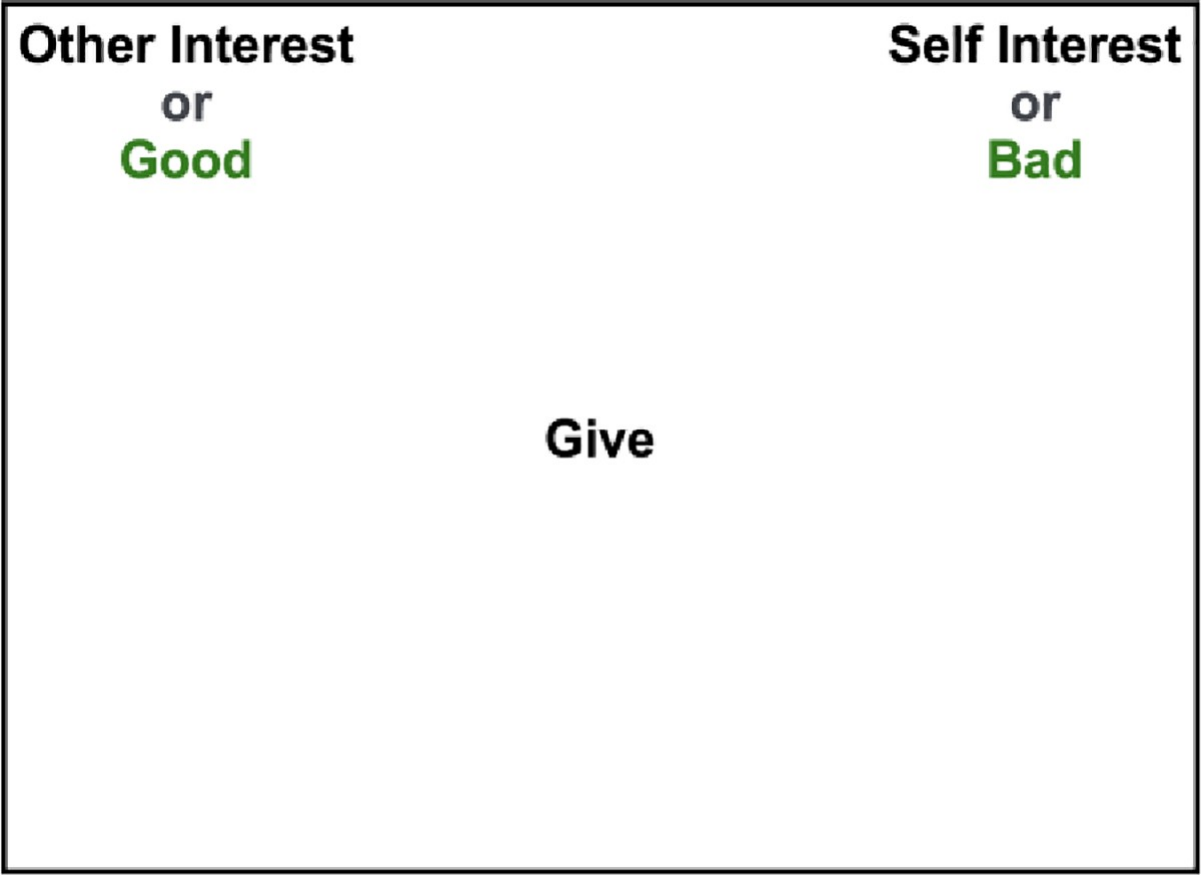
Sample categorization shown to participants in the prosocial compatible block of the SOI-IAT.

Following the practice blocks, participants completed two critical blocks. Critical blocks can be conceptualized as a combination of the practice blocks wherein participants classify both the pleasant/unpleasant words and the concept-related words into both categories (Good/Bad and Self-Interest/Other-Interest) at the same time. Importantly, in the critical blocks, both of the target concepts are displayed together. For instance, Self-Interest/Bad may be in the top left corner and Other-Interest/Good in the top right corner. Then, both the concept-related and pleasant/unpleasant words were presented randomly and individually in the middle of the screen, and participants classified them into the correct target category as fast as possible (Fig 1). There are two different critical blocks, described below.

*Prosocial compatible critical block*. In the prosocial compatible critical block, “Other-Interest” and “Good” are displayed in the same corner of the screen, and participants were tasked with categorizing pleasant and other-interest related concept words into the same category. In this block, a link is formed between the concepts of pleasantness and other-interest. As such, faster responses in this block suggest a positive implicit attitude toward other-interest, indicating higher prosociality.

*Prosocial incompatible critical block*. In the prosocial incompatible critical block, “Self-Interest” and “Good” are displayed in the same corner of the screen, and participants categorized both self-interest related words and pleasant words into the same category. Thus, in this block, a link is formed between pleasantness and self-interested. Faster responses in this block suggest a positive implicit attitude toward self-interest.

The SOI-IAT’s internal reliability in this study was *r =* 0.92. After completing the SOI-IAT, participants completed the scales below.

#### Explicit measures of prosociality

To investigate the relationship between implicit attitudes captured by the SOI-IAT and explicit measures of prosociality, participants completed a series of tasks and measures of explicit prosocial attitudes in the following order.

*Altruistic Personality Scale*. The Altruistic Personality Scale (APS; [13]) was used to determine whether the SOI-IAT was related to trait altruism by assessing the frequency with which participants reported engaging in altruistic behaviour. This 20-item, 5-point Likert scale measure includes such items as “I have given money to charity” rated from “1-Never” to “5-Very Often”. In this study, the APS scale displayed an internal reliability of Cronbach’s α = 0.89.

*Prosocial Personality Battery*. The Prosocial Personality Battery (PSB; [14]) was used to investigate the association of the SOI-IAT with existing measures of prosociality by assessing participant’s explicit prosocial attitudes. Only items related to Other-Oriented Moral Reasoning (MR; 6 items) and Social Responsibility (SR; 7 items) were used in the present research. This scale totals 13 items and includes “My decisions are usually based on concern for others” (MR) and “I would feel less bothered about leaving litter in a dirty park than a clean one” (SR). Possible responses ranged from “1-Strongly Disagree” to “5-Strongly Agree”. In the current study, the SR items displayed an internal reliability of Cronbach’s α = 0.84, and the MR items displayed an internal reliability of Cronbach’s α = 0.68.

*Interpersonal Reactivity Index*. The Perspective Taking (PT) and Empathic Concern (EC) sub-scales of the Interpersonal Reactivity Index (IRI; [18]) were used to investigate the SOI-IAT’s association with empathy. The EC and PT sub-scales were chosen because of their relevance to the present research, as the PT subscale measures an individual’s ability to take another’s perspective, and the EC subscale measures other-oriented empathy [18]. The PT and EC sub scales are both comprised of 7-items and on 5-point Likert scales ranging from 1- “Does describe me well” to 5- “Does not describe me well”. The PT subscale includes items such as “I try to look at everybody’s side of a disagreement before I make a decision”, whereas the EC subscale includes items such as “I am quite touched by things that I see happen”. In this study, the PT subscale displayed an internal reliability of Cronbach’s α = 0.49, and the EC subscale displayed an internal reliability of Cronbach’s α = 0.54.

*Hypothetical Dictator Game*. Participants reported how they would divide an imaginary $10 sum between themselves and an unpaid, unnamed other person. The distribution of funds had to equal $10. The amount of money participants allotted to the recipient represented a continuous measure of explicit prosocial behaviour.

After these scales, participant completed a number of exploratory measures that are not central to this paper, but are detailed on our OSF page for brevity and transparency.

#### Demographics, debriefing, and payment selection

Participants provided their demographic information (age, gender, approximate household income). In addition, participants noted their blood donation history by (1) how many years they had been donating blood, and (2) how many times they had donated blood in the past 12 months. Finally, participants were debriefed, thanked, and reimbursed for their time. Critically, participants could choose to claim their reimbursement as a $10 Starbucks gift card or donate $10 to Canadian Blood Services instead, which we considered a behavioural measure of prosociality. This study was approved by both the Simon Fraser University Research Ethics Board (2016s0118) and Canadian Blood Services Research Ethics Board (2017–044).

### Hypotheses

#### Convergent validity

As noted in our pre-registration, we hypothesized that SOI-IAT scores would be significantly and positively correlated with explicit measures of prosociality. Importantly, our pre-registration notes that while we predicted positive correlations, we did not expect exceptionally high correlations (*r* > 0.60) because past research has shown that implicit-explicit measures of the same construct often do not exhibit strong correlations ([5]; who found that the strength of implicit-explicit correlations is moderated by self-presentation effects, distinctiveness of the attitude from the norm, attitude strength, and attitude structure).

#### Predictive validity

As noted in our pre-registration, we hypothesized that SOI-IAT scores would correlate positively with participant’s choice to donate their $10 remuneration to Canadian Blood Services as opposed to claiming a $10 Starbucks card.

#### Construct validity

Finally, as noted in our pre-registration, we hypothesized that SOI-IAT scores would correlate positively with blood donation frequency. This is because we assumed that more prosocial blood donors would donate blood more often.

### Results

In order to conduct analyses, SOI-IAT scores were calculated using Greenwald, Nosek, & Banaji’s [16] improved IAT scoring algorithm (see OSF page), and we did not penalize incorrect responding. In the following analyses, the probability of a Type 1 error was 5% (α = 0.05, one-tailed for all analyses unless otherwise specified), and power (calculated post-hoc) was sufficient (82%) to detect moderate correlations (*r =* 0.23).

#### Convergent validity

Our first pre-registered hypothesis investigated the convergent validity of the SOI-IAT by examining whether SOI-IAT scores were positively correlated with existing explicit measures of prosociality. Correlation analyses (α = 0.05, one tail) revealed that *d-*scores were positively but not significantly correlated with existing explicit measures of prosociality assessed here using the Altruistic Personality Scale (*r*(151) = 0.07, *p =* 0.20), Social Responsibility subscale (*r*(151) = 0.13, *p =* 0.05), Moral Reasoning subscale (*r*(151) = 0.09, *p =* 0.14), Perspective Taking subscale of the IRI (*r*(151) = 0.09, *p =* 0.14), Empathic Concern subscale of the IRI (*r*(151) = 0.07, *p =* 0.21), and amount of money given to the stranger in the hypothetical dictator game (*r*(151) = 0.03, *p =* 0.36; see Table 1). Therefore, while associations were in the predicted direction, our hypothesis that SOI-IAT scores would be significantly positively correlated with explicit measures of prosociality received little support.

**Table 1:** Means, standard deviations, and the bivariate correlations between SOI-IAT d-scores and explicit prosociality measures in Study 1.

	SOI-IAT *d-*score	APS	MR	SR	EC	PT	Dictator Game
M (*SD*)	.14 (.57)	3.36 (.61)	3.95 (.58)	3.76 (.60)	3.63 (.50)	3.53 (.46)	6.50 (2.82)
SOI-IAT *d*-score	1						
APS	.07	1					
MR	.09	.18*	1				
SR	.13	.42*	.27*	*1*			
EC	.07	.36*	.34*	.45*	1		
PT	.09	.12*	.29*	.29*	.40*	1	
Dictator Game	.03	.17*	.14*	.26*	.15*	.13	1

*N =* 153. APS: Altruistic Personality Scale, MR: Moral Reasoning, SR: Social Responsibility, EC: Empathic Concern, PT: Perspective taking.

*p< .05, one-tail.

#### Predictive validity

Our second pre-registered hypothesis investigated the predictive validity of the SOI-IAT by exploring whether SOI-IAT scores were positively associated with blood donation frequency. Correlation analyses (α = 0.05, one tail) revealed that this correlation was positive but not significant, *r*(151) = 0.04, *p =* 0.32. Thus, while the relationship was in the predicted direction, findings did not support our hypothesis.

In light of recent but mixed evidence showing that women may be more inclined towards prosocial attitudes than men [19–21], we also explored whether gender moderates the relationship between SOI-IAT scores and explicit measures of prosociality. Using a series of linear regressions, we found that this was not the case in our data. Specifically, we did not observe significant interactions between SOI-IAT scores and gender when predicting explicit prosociality in this sample (APS: β = .08; *p* = .35; PSB SR: β = -.03; *p* = .77; PSB MR: β = -.01; *p* = .93; IRI PT: β = -.14, *p* = .10; IRI EC: β = -.06; *p* = .46).

#### Construct validity

Our final pre-registered hypothesis investigated the construct validity of the SOI-IAT by examining whether SOI-IAT scores were positively correlated with prosocial behaviour in participant’s choice to have their $10 reimbursement donated to Canadian Blood Services (*n* = 100; coded as 2) rather than claiming the $10 Starbucks gift card (*n* = 53; coded as 1). A point biserial correlation (α = 0.05, one tail) revealed that scores on the SOI-IAT were not significantly correlated with increased prosocial behaviour in this task, *r*(151) = -0.10, *p* = 0.12 and, if anything, were opposite to predictions.

### Discussion

Taken together, we did not find strong support for the validity of the SOI-IAT amongst a sample of Canadian blood donors but most associations were in the predicted direction. However, it is possible that SOI-IAT did not predict explicit scales and behavior in Study 1 because the blood donors sample was small (*n* = 153) and rather generous. As such, we conducted a similar study with a nationally representative panel of Americans to investigate the validity of the SOI-IAT amongst a larger and more diverse sample.

## Study 2: National panel

### Methods

#### Participants

We collected responses from 500 Americans representative of the US population in terms of gender, age, and income in exchange for $5 USD using Qualtrics Online Sample in January 2018. Of the 500 participants, a total of *n =* 33 respondents were excluded in accordance with pre-registered exclusion criteria: *n =* 32 were excluded because they were judged to be button-mashing, and *n =* 1 was excluded because they reported speaking English for less than 5 years. The latter pre-registered exclusion criterion was set because the SOI-IAT is a linguistic based reaction time measure in which results may be skewed if English language proficiency is low.

After exclusions, a sample of 467 participants remained (*M*_age_ = 54.4, *SD* = 15.1, range = 18–83, *n* = 243 female, *n* = 223 male, *n* = 1 other). This study was approved by the Simon Fraser University Research Ethics Board (2018s0070). The full pre-registration listing hypotheses, the data analysis plan, and exclusion criteria can be found on the OSF page here.

#### Procedures

Study 2 was similar to Study 1 with a few minor changes. First, because online data collection can sometimes lead to lower quality data, participants completed a one-item data quality check were asked “Do you commit to thoughtfully providing your best answers in this survey?” Participants could select “I will provide my best answers”, “I will NOT provide my best answers” or “I can’t promise either way”. If participants selected the second or third options (“I will NOT provide my best answers”, or “I can’t promise either way”), they did not complete the survey. Participants who selected the first response proceeded to the survey, which began with the same one-item trait happiness question and SOI-IAT as in Study 1 (detailed above). In this sample, the SOI-IAT had an internal reliability of *r =* 0.94.

#### Explicit measures of prosociality

After the SOI-IAT, participants completed the same several explicit measures of prosociality including the hypothetical dictator game, the Altruistic Personality Scale (Cronbach’s α = 0.90), the Prosocial Personality Battery Social Responsibility (Cronbach’s α = 0.68) and Moral Reasoning subscales (Cronbach’s α = 0.83), the Interpersonal Reactivity Index Perspective Taking (Cronbach’s α = 0.76) and Empathic Concern subscales (Cronbach’s α = 0.83), detailed above.

#### Demographics

Participants reported their demographics on a number of questions, including their age, gender, and household income.

#### Donation tasks

In Study 2, we investigated the predictive validity of the SOI-IAT with two real-life donation tasks.

In the first donation task, participants were provided with the option to give some of their time to help a fictional child in need. In particular, participants were presented with a letter from Connor, a fictional 8-year-old child who was undergoing treatment for cancer. Participants were led to believe that Connor was real, and were told that they could take some time to compose a response letter for Connor, should they choose to. Connor’s full note can be read on the OSF page. Examples of participant letters include “*I really hope it* [the treatment] *works for you too*. *Be brave and good things will happen*”, and “*Dear Connor*, *I feel horrible about your disease*. *Hang in there and best of luck with your upcoming treatment*. *I know you will beat this and start feeling better soon*!” The online survey recorded the amount of time that participants spent writing to Connor, which was operationalized as one measure of prosocial behaviour.

In addition to capturing the amount of time that participants spent writing to Connor, we also coded the content of their letters. To do so, five independent coders blind to hypotheses evaluated the extent to which the letter expressed empathy (1 –None to 4 –Extreme), support (1 –None to 4 –Extreme), degree of personalization (1 –None to 4 –Extreme), self-disclosure (1 –None to 4 –Extreme), assistance or advice (1 –None to 4 –Extreme), and a willingness to connect with Connor (1 –None to 4 –Extreme). In addition, coders rated whether the letter was coherent (0 –No, 1 = Yes). Average inter-rater reliability was IRR = 0.91 (range = 0.77–0.99). See our OSF page for the full coding scheme.

In a second donation task, participants were told that they had earned a bonus $1 USD in addition to their base pay from Qualtrics. To encourage feelings of ownership over this bonus, participants were asked to provide a personal code (including letters from their name, zip code, etc.) in place of their signature on a receipt to claim the $1. Having claimed their bonus, participants were given the option to donate none, some, or all of the $1 to the American Cancer Society. Participants indicated how much they would like to donate by selecting the number of cents on a 0–100 sliding scale. Amount donated was operationalized as an additional measure of prosocial behaviour.

Participants also completed a number of exploratory measures relevant to a separate research project. See our OSF page for a brief description of these items.

#### Debrief

Participants were debriefed and reimbursed for their participation. Payment included their base wage and whatever proportion of the $1 bonus they had chosen to keep.

### Hypotheses

#### Convergent validity

As noted in our pre-registration we hypothesized that SOI-IAT scores would correlate positively with explicit measures of prosociality. Again, while we expected positive correlations, we did not expect particularly large correlations for reasons detailed in Study 1.

#### Predictive validity

As noted in our pre-registration, we hypothesized that SOI-IAT scores would correlate positively with actual instances of prosocial behaviour assessed in two donation tasks. The first of these tasks was how long participants spent writing a response to a letter from Connor, a fictional youth with cancer whom participants were led to believed was real. As pre-registered, content of participant’s responses to Connor’s letter was also examined. The second donation was financial in nature, wherein participants were given the option to divide $1 between themselves and the American Cancer Society in one-cent increments.

### Results

In the following analyses, power (calculated post-hoc) was sufficient (<99.99%) to detect moderate correlations (*r =* 0.50). As in Study 1, *d*-scores were calculated using Greenwald, Nosek, & Banaji’s [16] improved IAT scoring algorithm (see OSF page).

#### Convergent validity

Consistent with our pre-registered analysis plan, we first assessed the convergent validity of the SOI-IAT by examining whether SOI-IAT scores were positively correlated with explicit measures of prosociality. Correlation analyses with α set to 0.05, one-tailed revealed that SOI-IAT *d-*scores were not correlated with explicit prosociality captured using the APS (*r*(465) *=* 0.05, *p =* 0.17), SR (*r*(465) *=* 0.07, *p =* 0.08), PT (*r*(465) = 0.05, *p =* 0.12), and amount of money given to the stranger in the hypothetical dictator game (*r*(465) *=* 0.03, *p =* 0.25; see Table 2 for full correlation matrix). There were, however, a few exceptions; the SOI-IAT was weakly correlated with the MR subscale, *r*(465) *=* 0.09, *p =* 0.02, and the EC subscale, *r*(465) *=* 0.09, *p =* 0.03. Therefore, for the most part, the data did not support our hypothesis that SOI-IAT scores would be positively correlated with explicit measures of prosociality, although correlations were in the predicted direction.

**Table 2:** Means, standard deviations, and the bivariate correlations between SOI-IAT d-scores and explicit prosociality measures in Study 2.

	SOI-IAT *d-*score	APS	MR	SR	EC	PT	Dictator Game
M(*SD*)	.00 (.56)	2.91 (.68)	3.72 (.56)	3.64 (.59)	3.80 (.68)	3.53 (.59)	4.59 (2.47)
SOI-IAT *d*-score	1						
APS	.05	1					
MR	.09*	.35*	1				
SR	.07	.21*	.29*	1			
EC	.09*	.32*	.59*	.46*	1		
PT	.05	.27*	.54*	.34*	.58*	1	
Dictator Game	.03	.12*	.31*	.27*	.30*	.21*	1

*N =* 457. APS: Altruistic Personality Scale, MR: Moral Reasoning, SR: Social Responsibility, EC: Empathic Concern, PT: Perspective taking.

*p< .05, one-tail.

As in Study 1, we also explored whether the relationship between SOI-IAT scores and explicit measures of prosociality was moderated by gender. Once more, a series of linear regressions revealed little support for this hypothesis in our sample (APS: β = .01; *p* = .85; PSB SR: β = .03; *p* = .56; PSB MR: β = .02; *p* = .73; IRI PT: β = .05, *p* = .29; IRI EC: β = .-04; *p* = .31).

#### Predictive validity

Consistent with our pre-registered analysis plan, we also examined the predictive validity of the SOI-IAT by investigating whether SOI-IAT scores were positively correlated with prosocial behaviour as assessed in the two real-life donation tasks.

First, we conducted correlational analyses with α set to 0.05, one tail, to investigate whether SOI-IAT scores were associated with prosocial behaviour in response to Connor’s note. Analyses revealed that there was a small but significant positive correlation between SOI-IAT scores and coder ratings of support in participant’s responses to Connor’s letter, *r*(465) *=* 0.09, *p* = 0.04. Additional analyses revealed that scores on the SOI-IAT were not correlated with other dimensions of prosocial note content (see Table 3), or how long participants spent composing their response to Connor (*r*(421) = -0.03, *p =* 0.26). We were unable to examine whether SOI-IAT scores predicted levels of self-disclosure and a desire to connect with Connor because ratings of these dimensions revealed substantial floor effects with 88% and 94% of participants scoring the lowest values, respectively.

**Table 3:** Means, standard deviations, and bivariate correlations between SOI-IAT d-scores and coder evaluations of participant responses to Connor’s letter in Study 2.

	SOI-IAT *d*-score	Time	Coherence	Mistake	Empathy	Support	Personal	Assistance
			(α = .99)	(α = .89)	(α = .89)	(α = .90)	(α = .94)	(α = .77)
M(*SD*)	.00(.56)	103.06 (131.55)	.77(.41)	.33(.50)	1.48(.60)	2.57 (.65)	1.94(.73)	1.76(.46)
SOI-IAT *d*-score	1							
Time	-.03	1						
Coherence	.04	*-*.03	1					
Mistake	-.06	*-*.01	.09*	1				
Empathy	.04	.08	.07	.03	1			
Support	.09*	.01	.28*	.18*	.37*	1		
Personal	.03	.03	.22*	.28*	.51*	.63*	1	
Assist	*-*.02	*-*.04	.23*	.16*	.14*	.32*	.31*	1

*N =* 457. Mistakes: Number of Mistakes, Personal: Personalization, Assistance: Offers of Personal Assistance or Advice.

*p< .05, one-tail.

Second, we conducted correlational analyses with α set to 0.05, one tail, to investigate whether SOI-IAT scores were associated with financial donations to the American Cancer Society. Analyses revealed that SOI-IAT scores were not significantly correlated with larger donations, *r*(465) *=* 0.04, *p* = 0.19. However, over half of the sample (238 of 467 respondents, 51.1%) donated the full $1 to American Cancer Society, meaning that the donation distribution was extremely skewed. To address this concern, we compared the SOI-IAT *d-*scores of participants who chose to donate over half of their $1 bonus (50–100¢, *n* = 343, *M =* 0.02, *SD* = 0.58) to the *d-*scores of participants who donated less than half of their $1 bonus (0–49¢, *n* = 124, *M =* -0.05, *SD* = 0.51) to the American Cancer Society. Post hoc power calculations indicated that we had 88.7% power to detect a moderate difference (*d =* 0.30) between the means. We predicted that the *d-*scores amongst participants who donated more than 50% of their bonus would be significantly higher, indicating more prosociality, than participants who donated less than 50% of their bonus. An independent samples *t*-test (α = 0.05, one tail) revealed these *d-*score means between high and low donors did not differ, *t*(465) = -1.32, *p =* 0.19. Thus, the data did not support our hypothesis that SOI-IAT scores would be associated with real life prosocial behaviour in this donation task.

### Discussion

In Study 2, we examined whether SOI-IAT scores were positively correlated with explicit measures of prosociality, and whether the SOI-IAT was capable of predicting two key forms of prosocial behaviour (financial and interpersonal assistance) amongst a large and diverse sample. Although SOI-IAT scores were generally associated with higher scores on explicit scales of prosociality and actual rates of prosocial behaviour, few of these relationships were statistically significant. Thus, the results of Study 2 provide minimal support for the validity of the SOI-IAT as an implicit measure of prosociality.

## Study 1 and 2 means comparison

As a final test of the construct validity of the SOI-IAT, we investigated whether the SOI-IAT could differentiate between the two distinct samples in Studies 1 and 2. Specifically, we compared the SOI-IAT scores from the blood donors sample in Study 1 (the prosocial sample) to the SOI-IAT reported by the representative sample of Americans in the Study 2 (the control sample) to assess whether the SOI-IAT could distinguish predictable differences in prosocial attitudes between samples.

Consistent with our pre-registration (see Hypothesis 1 in linked pre-registration), this analysis included only regular blood donors, defined as having donated blood at least 3 times in the past 12 months—half as many times as is allowed in 12 months by Canadian Blood Services [22]. This sub-sample of blood donors was comprised of 73 individuals (*M*_age_ = 43.7, *SD* = 16.3, range = 19–77, 47 male, 25 female, 1 preferred not to say). This sample was compared to the full national panel sample (*M*_age_ = 54.4, *SD* = 15.1, range = 18–83, 243 female, 223 male, 1 other).

### Hypothesis

We hypothesized that the average SOI-IAT score amongst regular blood donors (Study 1) would be significantly higher than the average SOI-IAT score reported in the nationally representative panel of Americans (Study 2).

### Results

To test our pre-registered hypothesis, we conducted an independent samples *t*-test with alpha set to 0.05, one-tail comparing the average *d*-score of regular blood donors from Study 1 to the average *d*-score of participants in the national panel from Study 2. Power, calculated post-hoc, was sufficient (80%) to detect a moderate difference (*d* = 0.32) between the means. Analyses revealed that the average *d*-score amongst regular blood donors (*M* = 0.14, *SD* = 0.54, 95% CI [0.01, 0.27]) was significantly higher than the average *d-*score amongst the control sample (*M* = 0.00, *SD =* 0.56, 95% CI [-0.05, 0.05], *t*(537) *=* 1.99, *p* = 0.02, *g* = 0.25, 95% CI [0.08, 0.45]). Thus, our hypothesis that SOI-IAT scores amongst the regular blood donors would be significantly higher than SOI-IAT scores amongst the control sample was supported.

## General discussion

The current research examined the construct validity of the SOI-IAT, a new measure of implicit prosociality, in several ways. First, we hypothesized that SOI-IAT scores would be positively correlated with scores on explicit measures of prosociality. This hypothesis was not strongly supported in either study, with a few notable but weak exceptions in Study 2. Second, we hypothesized that SOI-IAT scores would be positively correlated with real-life prosocial behaviour. With one exception in Study 2 (interpersonal support in a letter to a sick child), analyses revealed no such relationship in either study. However, SOI-IAT scores and associations with both explicit measures and prosocial behaviour were largely in the predicted direction in both studies. Finally, we hypothesized that the regular blood donors’ sample (Study 1) would demonstrate significantly higher other-interest on the SOI-IAT than the control sample (national panel of Americans, Study 2). This pre-registered hypothesis was supported, but the effect size was small. This suggests that the SOI-IAT may be able to detect some group differences.

Taken together, these results provide mixed but limited support for the SOI-IAT. While it seems to be able to distinguish predictable differences between samples, *d-*scores on the SOI-IAT were uncorrelated with several explicit measures of prosociality. This was not wholly unexpected given the low correlations typical of implicit-explicit measures [5], and explicit measure’s potential for bias due to self-presentation effects. However, the lack of observed relationship between the SOI-IAT scores and explicit measures of prosociality may also be explained by the design of the SOI-IAT (discussed in the limitations section below).

The SOI-IAT’s inability to predict behaviour may not be surprising because IATs are less likely to predict behaviour when they do not correlate strongly with explicit self-report measures [23]. Additionally, these null results may be explained by past research findings that IAT’s are better suited to detecting group-level rather than individual-level attitude differences [8]. This may explain why blood donor’s SOI-IAT scores indicated higher prosociality than the control sample, but in neither study did SOI-IAT scores predict individual’s prosocial behaviour.

### Limitations

Because the SOI-IAT is a new application of Greenwald and colleagues’ (1998) original Implicit Association Test (IAT), the common criticisms of the IAT merit mention. For instance, while Greenwald and Banaji [16] maintain that IAT measures implicit attitudes, skeptics contend that IATs may instead measure knowledge of cultural attitudes [24]. Alongside this, the IAT’s internal reliability and ability to predict relevant behaviour have been called into question [25–27]. Even so, despite these potential shortcomings, researcher’s enthusiasm for this test has not been diminished as illustrated by the continued development of new versions of the IAT (for instance, [28–30]). Thus, despite ongoing debates, the IAT is still a very commonly used measure of implicit attitudes. While this is not evidence for the validity of the IAT, and caution should still be exercised in making inferences based on this measure, the IAT seems to be the best implicit attitude measure available [31]. Further, it is worth noting that the IAT’s criticisms also apply to many frequently used psychological measures.

Beyond these overarching concerns of the IAT, the present research has some important limitations. For instance, Hoffman et al. [32] found that implicit-explicit correlations were particularly low when the concept categories were pronouns. Indeed, Hoffman et al. [32] specifically lists self vs. other (the categories used in the present research) as examples of problematic categories due to ambiguity regarding who “other” refers to. Thus, the low correlations between the SOI-IAT and explicit measures of prosociality may be related to our choice of concept categories and explicit measures of prosociality, rather than reflecting the convergent validity of the SOI-IAT. Future research may build upon this limitation by re-programming the SOI-IAT using non-pronoun target categories. Hoffman et al. [32] suggests using specific, un-ambiguous categories, so perhaps the target categories “Own Benefit” or “Stranger’s Benefit” are worth consideration.

In addition, future research may build on the present work by selecting less demanding explicit prosociality scales. Hoffman et al. [32] found that implicit-explicit correlations were lower when explicit measures required effortful retrieval of information. In the present research, many of the explicit self-report measures ask participants to recall specific instances of past behaviours, requiring cognitive effort (for instance, the Altruistic Personality Scale asks, ‘How often do you do volunteer work for charity?’). Thus, our choice of explicit measures of prosociality may explain why we did not observe significant correlations between the SOI-IAT and explicit measures of prosociality. Future research could improve upon our design by using less effortful explicit self-report measures.

The SOI-IAT’s low predictive validity may be explained by a strong pressure to donate. In Study 1, for instance, the researcher verbally asked participants whether they wanted to claim a $10 Starbucks gift card or donate the $10 to Canadian Blood Services after completing the survey. Because this request was made in person after participants had recently completed scales that clearly assessed their generosity (i.e., “How often do you donate to charity?” from the Altruistic Personality Scale), participants may have felt pressured to donate their remuneration to Canadian Blood Services. In fact, 65.4% of participants donated their $10 remuneration (*n =* 100) instead of claiming the Starbucks gift card (*n* = 53). Similarly, in Study 2, participants were asked if they wanted to donate some of a monetary bonus to the American Cancer Society after reading a note from a child fighting cancer. Perhaps not surprisingly, the majority of participants (51.1%) donated the full amount. These design choices may have inflated donation behaviour, which restricted variability and masked a relationship between SOI-IAT scores and donation behaviour. For this reason, we recommend that future research take steps to avoid strong demand characteristics, perhaps by asking participants for their donation choice early on, anonymously, and before explicit measures of prosociality.

Along similar lines, past research has found that IAT responses may be swayed by the context in which they are taken [33]. Specifically, in Study 1, the SOI-IAT was administered in Canadian Blood Services immediately after participants donated blood. Because blood donation is a prosocial behaviour which Canadian Blood Services celebrates (i.e., giving donors “I saved a life today” stickers), receiving positive feedback for engaging in a prosocial behaviour immediately before taking the SOI-IAT may have caused participants to display inflated positive attitudes toward prosociality, regardless of their underlying implicit attitude. Thus, the differences in prosociality between the regular blood donors and national panel samples may have been exaggerated. Future research may overcome this limitation by replicating Study 1 in an environment that does not have the potential to prime prosocial orientations. For instance, perhaps blood donors could be given a link to the SOI-IAT survey after donating blood, which they would be instructed to complete from home a week later.

Finally, it is worth stating that the samples in Study 1 and Study 2 were drawn from different countries. As such, it is possible that features other than implicit prosociality (e.g., cultural norms, national identity, recent events) explain group differences in SOI-IAT scores. Thus, our finding that regular blood donors exhibited higher implicit prosociality than the control sample may be confounded by some unknown factor. Future research would do well to either draw the compared samples from the same population, or match the samples based on theoretically relevant information in order to avoid potential confounds.

### Applications

With necessary refinement, the SOI-IAT’s construct validity could have important implications for research on prosociality. In particular, if a controlled reaction time measure like the SOI-IAT were able to detect some predictable differences in implicit prosociality it would suggest that prosocial attitudes may be fundamental traits underlying the variability in human’s treatment of others. This may also shed light on the origins of such attitudes, as Greenwald and Banaji ([9]) suggest that implicit attitudes are rooted in past experience. However, further research is required before this can be confirmed.

### Implications

Our findings also provide some insight into the broader challenges of adapting and creating new implicit attitude measures. In particular, while the SOI-IAT was able to detect group differences between the prosocial and control samples (but not small differences in prosocial behaviour), the explicit measures of prosociality were just as effective in distinguishing these groups, and such measures’ ability to predict behaviour has been well established [13, 34, 35]. This is not to suggest that IATs or other implicit measures hold no value; implicit measures remain most effective at circumventing self-presentation effects, and it is likely that we did not measure those behaviours that are best predicted by implicit attitude measures [i.e., 32]. However, when creating and validating new implicit attitude measures, it is worth considering the challenges of selecting outcome variables that tap implicit processes.

More broadly, our research highlights the importance of publishing null results. We undertook the present studies in light of past work suggesting that gut level reactions to self- vs. other-interest content may provide novel insight into unbiased prosocial character. Yet, our data provide mixed support for this possibility and a few underlying theoretical predictions. Being as the field has recently recognized that sharing null and mixed results is vital to scientific progress (Transparency and Openness Promotion Committee guidelines; [36]), we hope that including these data in the scientific record can assist with future meta-analyses and provide researchers with a more complete and accurate picture of the science of implicit processes. We further hope that our experience encourages other researchers to “empty the file drawer” and publish null or mixed results for the sake of scientific progress.

## Conclusion

The present research offers mixed support for a new measure of implicit prosocial attitudes called the SOI-IAT. As hypothesized, regular blood donors displayed significantly higher levels of other-interest (vs. self-interest) as captured on the SOI-IAT than the control sample. On the other hand, SOI-IAT scores were not significantly correlated with the majority of explicit measures of prosociality or real-life prosocial behaviour in either study, although most associations were in the predicted direction. While these findings are promising, future research should build upon the limitations of the present research in hope of a more robust validation of the SOI-IAT.
